# Synthesis and Characterization of an Novel Intercalated Polyacrylamide/Clay Nanocomposite

**DOI:** 10.3390/gels9020104

**Published:** 2023-01-24

**Authors:** Song Zhang, Falin Wei, Pingde Liu, Cheng Cai, Zhihui Zeng, Zhentao Yu, Zengbao Wang, Yingrui Bai

**Affiliations:** 1 Research Institute of Petroleum Exploration and Development, PetroChina, Beijing 100083, China; 2 Key Laboratory of Oilfield Chemistry of CNPC, Beijing 100083, China; 3 School of Petroleum Engineering, China University of Petroleum (East China), Qingdao 266580, China; 4 Beijing Key Laboratory of Unconventional Natural Gas Geology Evaluation and Development Engineering, China University of Geosciences (Beijing), Beijing 100083, China

**Keywords:** intercalated polymer, synthesis, salt resistance, thermal stability

## Abstract

Solving the problem of the low temperature and low salt resistances of conventional polyacrylamide and the high cost of functional monomers, and thus, introducing it to the interlayer space provided by a layered structure for polymer modification, is a promising option. In this study, montmorillonite was used as the inorganic clay mineral, and an intercalated polyacrylamide/clay nanocomposite was synthesized via in situ intercalation polymerization. The optimal synthesis conditions were a clay content of 10.7%, preparation temperature of 11 °C, initiator concentration of 2.5 × 10^−4^ mol/L, and chain extender concentration of 5%. The IR results showed that the polymer was successfully introduced to the nanocomposite. The synthesized intercalated polyacrylamide/clay nanocomposite exhibited a better thickening effect, good viscoelasticity, and better salt resistance and thermal stability than polyacrylamide. In addition, the thickening capacity and thermal stability were superior to the salt-resistant polymer, with a 16.0% higher thickening viscosity and a 15.1% higher viscosity retention rate at 85 °C for 60 d. The intercalated polyacrylamide/clay nanocomposite further expanded the application of polyacrylamide in petroleum exploitation.

## 1. Introduction

Among the most frequently used synthetic polymers in petroleum exploitation, polyacrylamide has been widely applied in production processes, such as tertiary oil recovery, water shutoff, profile control, fracturing, and water treatment [1,2]. However, amide groups in polyacrylamide undergo rapid hydrolysis under acidic or alkaline conditions. Moreover, the hydrolysis rate increases rapidly with temperature increases, which lowers the temperature and salt resistances of polyacrylamide. Hence, polyacrylamide is unsuitable in high-temperature and high-salt oil reservoirs. In addition, under low-temperature and high-salinity conditions, the polymer solution in oilfield-produced water shows poor thickening capability, restricting its large-scale applications.

Researchers applied graft co-polymerization to improve the temperature and salt resistance and introduced functional monomers. Consequently, various functional polymers were designed and prepared, including amphoteric polymers [3,4], temperature- and salt-resistant copolymers [5,6], hydrophobic associative polymers and multi-component combined (complex) copolymers [7,8], and blend polymers and comb polymers [9,10]. These polyacrylamide-based polymers display improved temperature and salt resistances; however, introducing functional monomers results in higher production costs, thus, decreasing the polyacrylamide market.

Different modification strategies were proposed to introduce the polymer in the interlayer space of layered structures. Clay-based materials were first proposed in the 1980s by Okada et al. at the Toyota Research Center in Japan [11]. The polymerization in the clay interlayer space was applied to design several polymer-based organoclays. For instance, nylon 6/montmorillonite nano-hybrid material contains an organic polymer and an inorganic phase layered clay [12,13,14,15]. Importantly, the preparation of organoclays generates high-performance polymer materials [16].

Shengjie and Zongneng et al. [17] studied polystyrene (PS)/montmorillonite composite, which was prepared using a melt intercalation method. However, the organic soil layers’ organic groups interact with the PS, favoring its location in the montmorillonite layered silicate. Jiankun [18,19] prepared an epoxy resin/montmorillonite nanocomposite. The formation mechanism and properties of the composite material were studied, suggesting that the clay exfoliation was independent of the curing temperature. Giannelis [20] reported that an epoxy resin nanocomposite containing 4% layered silicate exhibited glass-transition behavior and a storage modulus that was 60% higher than the ordinary epoxy resin. In the elastomeric state, the storage modulus was 450% higher than that of ordinary epoxy resin. However, the change in the nanocomposite material moduli was insignificant, even at 10% clay content. Pinnavaia [21,22,23] found that the modulus and strength of epoxy resin nanocomposite increase by 10 times at the glass transition temperature. In the epoxy resin/montmorillonite, the montmorillonite is first converted into lipophilic clay, solving the shrinkage problem in the curing of epoxy resin. Guohua [24] and Dietsche [25] studied acrylic/montmorillonite nanocomposite, where the acrylic resin’s mechanical properties and heat resistance improved. Moreover, the air-tightness of the resin film or coating was strengthened, and the combustion and water resistances were enhanced. In addition, plasticizer migration was also effectively inhibited by mixing with polyvinyl chloride (PVC).

A polyester/montmorillonite nanocomposite is the typical material prepared using in situ intercalation polymerization [26]. The polymerization method is either direct esterification or transesterification; organic alcohol amines or amino acids polymerize with the intercalated montmorillonite. They are added to the reactor and mixed evenly to form a melt-through transesterification or esterification reaction. Polyester/clay nanocomposites are obtained after further polycondensation under a vacuum.

In addition to the conventional polymer and montmorillonite nanocomposites, inorganic particles and montmorillonite, such as silica/montmorillonite (MMT) nanocomposites [27], can also be compounded using intercalation.

## 2. Results and Discussion

### 2.1. Synthesis Mechanism of the Intercalated Polymer

Montmorillonite was chosen as the inorganic clay mineral for intercalation in this study. The interlayer spacing was about 1 nm, with a negatively charged surface. When cations are adsorbed on the layers, they neutralize the surface. Based on the Na^+^ and Ca^2+^ cationic exchange in the interlayer space of the clay, the organic cations enter this gallery, increasing the number of layers. In addition, the organic cations change the polarity of the inorganic clay mineral layer and reduce the surface energy.

**Figure 1:**
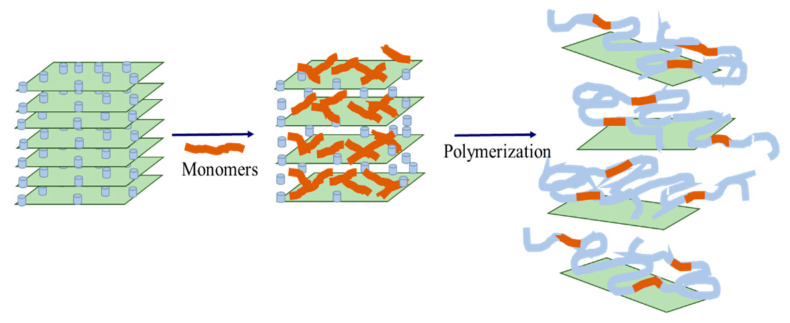
Schematic diagram of in situ intercalated polymerization.

The intercalated polymers are synthesized via in situ intercalation polymerization (monomer intercalation method, Figure 1). First, the modified layered silicates are dispersed in a monomer solution. The monomer partially enters the interlayer spacing, polymerizes, and increases the interlayer space. In addition, the in situ polymerization releases a large amount of heat (ΔH < 0), thus, exceeding the effect of ΔS. Accordingly, the layer space rapidly extends, forming an intercalated or exfoliated intercalated polymer. The formed intercalated polymer is crosslinked using a crosslinking agent, such as organic chromium, to prepare for the intercalated polymer gel, steering the flow direction of existing deep liquid and enhancing the low strength and poor stability of regulating and driving systems. At the same time, the material cost is reduced since inorganic clay minerals replace some of the monomers needed for synthesizing the polymer.

### 2.2. Optimization of the Synthesis Conditions for the Intercalated Polymer/Clay Nanocomposite

#### 2.2.1. Effect of the Inorganic Clay Mineral Content

**Figure 2:**
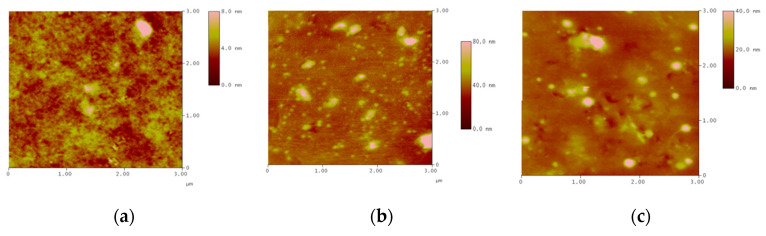
Inserted layer polymer contrast of different concentrations of inorganic clay mineral content: (**a**) 3.85%; (**b**) 10.7%; (**c**) 16.7%.

Different concentrations of montmorillonite clay (mass concentrations of 3.85, 10.7, and 16.7%) were mixed with the monomer to produce the intercalated polymer. The surface morphology of nanocomposite was investigated using an atomic force microscope in three dimensions (the horizontal X-Y plane and the vertical Z dimension). Figure 2 shows that the increase in the clay content caused an obvious agglomeration. At a clay content of 10.7%, the polymer was more uniformly distributed; only small particles were relatively well dispersed without any agglomeration. When the clay content reached 16.7%, the polymer’s homogeneous distribution was lost, large agglomerates formed, and many inorganic particles with a low degree of exfoliation were present. With the increase in montmorillonite content, the agglomeration increased, which was mainly caused by the increase in concentration; the decrease in the average distance of molecular chains (the nearest molecular position); and the increase in electrostatic force, van der Waals force, and other interactions between particles. These results indicated that the inorganic mineral clay consisted of small and highly dispersed particles. Therefore, the clay content should not be higher than 10.7% in the preparation of the intercalated polymer.

#### 2.2.2. Effect of the Temperature

**Figure 3:**
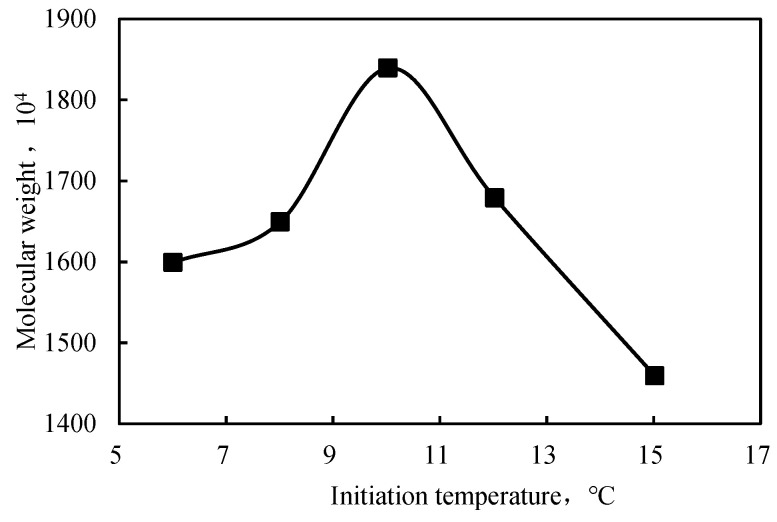
Effect of the initiation temperature on the molecular weight of the intercalated polymer.

The polymerization temperature affected the polyacrylamide formation process. When the temperature was too high, the free radicals decomposition rate was fast; hence, the polymerization rate was fast. The concentration of free radicals generated per unit of time and volume was also high, which increased the temperature. Therefore, the molecular weight of the polymer decreased and aggregation appeared. At low temperatures (≤10 °C), acrylamide was precipitated from the system. The molecular weight increased with temperature to a maximum value at 10 °C and then decreased (Figure 3). This behavior was explained by the activity of free radicals, which was low at relatively low temperatures and unfavorable for the chain transfer involved in the reaction of the free radicals. At higher temperatures, the rate of initiation increased; therefore, the resulting polymer had a lower molecular weight.

**Figure 4:**
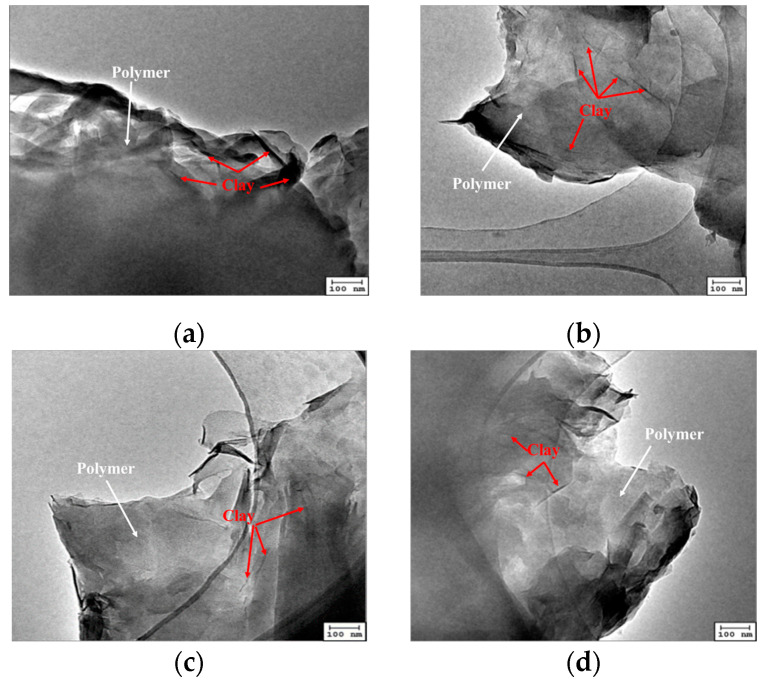
TEM images of the intercalated polymer synthesized under different initiation temperatures: (**a**) 8 °C; (**b**) 10 °C; (**c**) 11 °C; (**d**) 15 °C.

Figure 4 shows the transmission electron images of the intercalated polymer prepared at different temperatures (8, 10, 11, and 15 °C). The intercalated polymer/clay nanocomposites were mainly formed as thin sheets. After intercalation, a considerable part of the inorganic mineral clay was exfoliated, generating nanoscale sheets used as a skeleton by the polymer, connecting the sheets. The samples prepared at 8 and 10 °C showed few unexfoliated clay particles, which were not observed in the images corresponding to the samples synthesized at 11 and 15 °C. After the exfoliation, the inorganic mineral clay was dispersed in the composite material.

When the temperature increased in the experimental range, the viscosity and molecular weight increased and then decreased, reaching the highest values at 10 or 11 °C. Therefore, 11 °C was selected to prepare the intercalated polymer/clay nanocomposites.

#### 2.2.3. Effect of the Initiator Concentration

The initiator concentration impacted the polymer’s molecular weight, which tended to decrease as the initiator concentration increased. However, when the concentration was too low, the polymerization rate was slow, generating a polymer with a low molecular weight. When the initiator concentration was too high, the heat generated by polymerization could not be released, resulting in a low molecular weight. Therefore, a rational selection of the initiator concentration could obtain a high-molecular-weight polymer.

**Figure 5:**
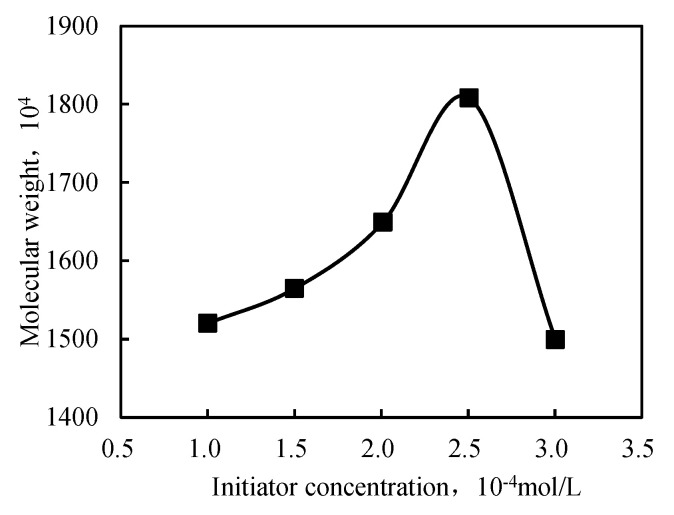
Relationship between the initiator concentration and molecular weight of the intercalated polymer.

**Figure 6:**
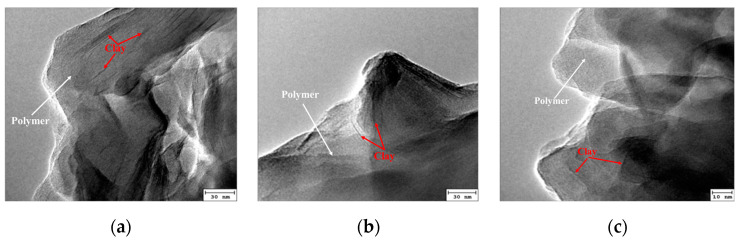
TEM images of the intercalated polymer synthesized under different initiator concentrations: (**a**) 1.0 × 10^−4^; (**b**) 1.5 × 10^−4^; (**c**) 2.5 × 10^−4^.

Figure 5 illustrates that the relative molecular weight increased and decreased with increasing initiator concentration. When the amount of initiator was small, due to the low initiator decomposition rate, the amount of free radicals was small. The slow polymerization reaction resulted in large polymer oligomer content and small polymer molecular weight. With the increase in the initiator amount, the polymerization rate gradually accelerated, the oligomer content in the polymer gradually reduced, and the molecular weight increased. Further increasing the amount of initiator increased the probability of multipoint polymerization and hindered the production of long molecular chain polymers; thus, the molecular weight decreased. The highest molecular weight was observed at a 2.5 × 10^−4^ mol/L initiator concentration. The corresponding viscosity had the highest value. This behavior can be explained in terms of primary radicals, which showed a higher concentration at a higher initiator concentration. Hence, the polymerization occurred at a high rate, favoring the formation of a short polymer chain. The morphology of the intercalated polymer was more defined when the initiator concentration increased, as displayed in Figure 6. The inorganic clay mineral was also uniformly dispersed in the intercalated polymer. However, as the initiator concentration exceeded 2.5 × 10^−4^ mol/L, the intercalated polymer/clay nanocomposite exhibited an irregular morphology. Hence, the optimal initiator concentration was found at 2.5 × 10^−4^ mol/L.

#### 2.2.4. Effect of the Chain Extender Concentration

**Figure 7:**
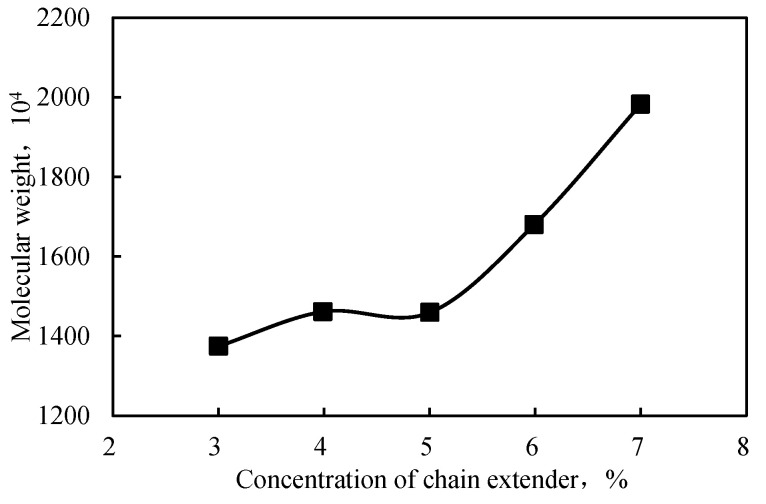
Relationship between the chain extender concentration and the polymer’s molecular weight.

A chain extender, which reacts with the functional groups on linear polymer chain to increase the molecular chain and the molecular weight, was used to synthesize the intercalated polymer given the development of a nanocomposite with a high molecular weight and improved thickening effect between the inorganic sheets of a clay mineral. The optimal concentration of the extender was established by screening different concentrations of LZJ-23, which is a compound with active hydrogen. The results are displayed in Figure 7 as a plot of molecular weight vs. extender concentration, highlighting the molecular weight increase with extender concentration. Accordingly, a molecular weight of almost 2000 Da was obtained at a 7 wt.% extender concentration.

**Figure 8:**
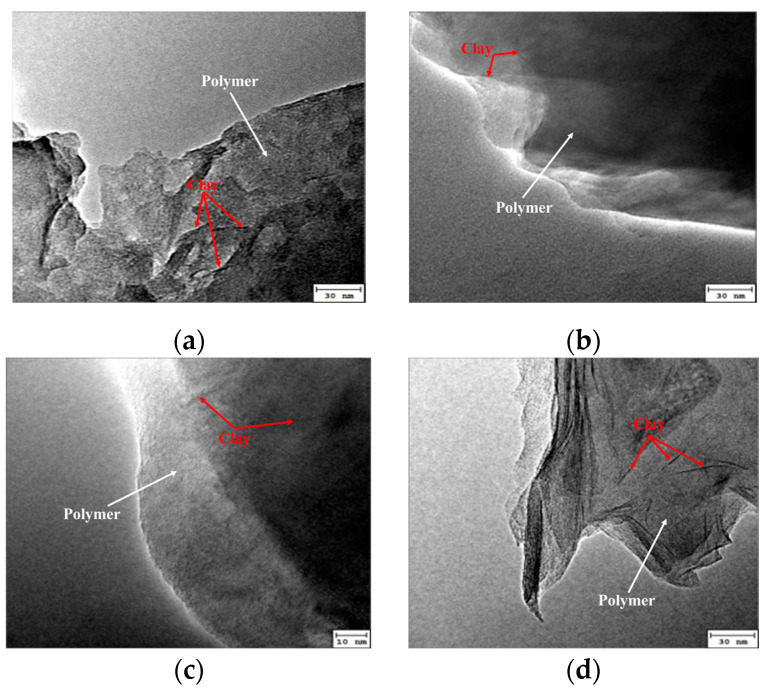
TEM of intercalated polymer synthesized by different concentrations of a chain extender: (**a**) 3%; (**b**) 4%; (**c**) 5%; (**d**) 6%.

Figure 8 illustrates the morphology of the intercalated polymer/clay nanocomposite prepared at different extender concentrations.

Figure 7 and Figure 8 reveal that the polymer’s molecular weight was lower at a lower concentration of the chain extender. Moreover, the morphological investigation showed that the exfoliation was incomplete when the extender concentration was 6%. Adding a higher concentration of chain extender produced a long-chain polymer. The resulting molecular size was too large and unconducive to enter the montmorillonite layer; thus, the peeling degree of the layer decreased. Therefore, the chain extender’s optimized concentration was considered to be 5%.

### 2.3. Physico-Chemical Properties of the Intercalated Polymer/Clay Nanocomposites

#### 2.3.1. FTIR Analysis

**Figure 9:**
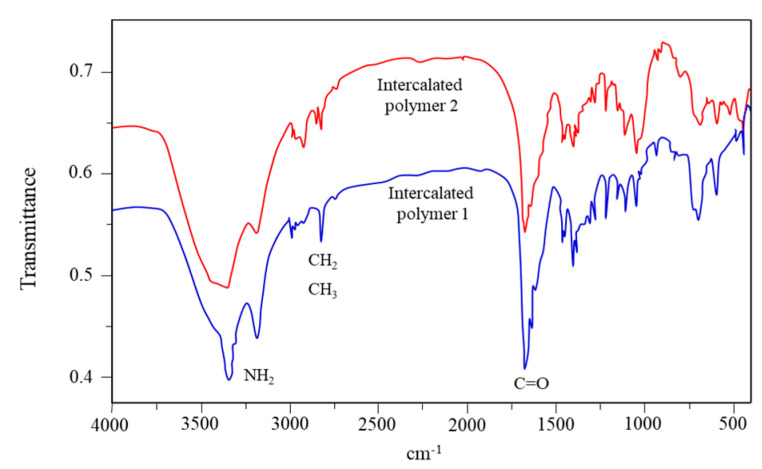
Infrared spectra of the intercalated polymers.

Figure 9 illustrates the FTIR spectrum of the intercalated polymer/clay nanocomposite. An obvious peak at 1670 cm^−1^ was observed for the polymer before and after the intercalation, which was attributed to the stretching vibration of the carbonyl group (C=O). The peaks at 3350 and 3200 cm^−1^ were attributed to the symmetric and asymmetric stretching vibrations of N-H in the polyacrylamide, respectively, suggesting that the polymer was successfully included in the nanocomposite.

#### 2.3.2. Thickening Effect

Under optimal conditions, the intercalated polymer in the organoclay prepared possessed better solubility, while the inorganic clay was more uniformly distributed, displaying a higher degree of exfoliation. At concentrations of 800 to 1600 mg/L , the average viscosities of polymer solutions increased by 48.9% and 16.0%, respectively. Therefore, the new nanocomposites’ thickening effect was attributed to the intercalated polymer and the exfoliated clay, synergistically increasing the viscosity. The viscosities of the intercalated, high-molecular, and salt-resistant polymers were compared under different salinity conditions, as shown in Table 1 and Table 2.

Table 1 shows that the intercalated polymer had a better thickening effect in clear water than the high-molecular-weight and salt-resistant polymers. The polymer intercalated into the montmorillonite layer and formed a secondary bond and physical cross-linking structure with three-dimensional laminates, indicating better viscosity-increasing characteristics. Table 2 shows that the salinity influenced the viscosity of the polymers. It was noticed that the polymer solution’s viscosity decreased as the water salinity increased. When the water salinity increased from 729.3 to 4012.7 mg/L, the intercalated polymer’s solution had the highest viscosity and the best salt resistance, even better than the salt-resistant polymer. The high-molecular-weight polymer had the lowest viscosity value and the worst salt resistance.

With the increased salinity, the cation concentration increased. Cations in water migrated to the Stern layer on the polymer surface, neutralizing part of the negative charge on the molecular chain surface, thereby decreasing the thickness of the polymer molecular diffusion layer, molecular chain curling and shrinking, and viscosity. However, the lamellar structure of the intercalated polymer exhibited greater rigidity and strength and strong anti-curling ability. In addition, due to the cation exchange property of montmorillonite, the lamellar surface had excess negative charges, neutralizing excess cations. Moreover, the viscosity of the intercalated polymer was less affected by the salinity.

#### 2.3.3. Viscoelastic Properties

**Figure 10:**
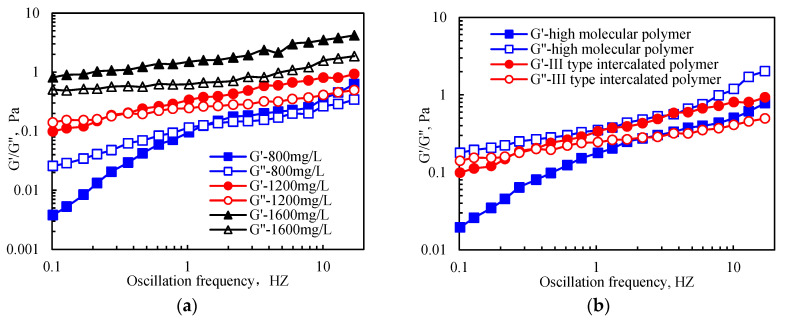
Viscoelasticity of the intercalated and high-molecular-weight polymers: (**a**) the III-type intercalated polymer of different concentrations; (**b**) 1200 mg/L III-type intercalated and high-molecular-weight polymers.

Figure 10 illustrates that under a certain range of oscillation frequencies, the intercalated and polyacrylamide polymers had a higher storage modulus (G’) and loss modulus (G′′) at higher polymer concentrations. In addition, a smaller frequency corresponding to the intersection of G′ and G′′ was observed at the highest polymer concentration. In contrast, the oscillation frequency corresponding to G′ = 0 was higher at higher polymer concentrations. The obtained results showed that the high oscillation frequency required for a polymer solution to lose its elasticity was high. In addition, at higher solution concentrations, the viscoelastic properties of the solution were improved. At the same concentration, the polymer solution had a dominant viscosity relative to the elasticity. At low oscillation frequency, the viscosity of the polymer solution was dominant, while the elasticity was not obvious. However, at high oscillation frequency, the increased deformation energy was absorbed by intramolecular or intermolecular elastic deformation; thus, the viscosity decreased and the elasticity dominated. When the oscillation frequency reached a certain level, the deformation energy was not completely absorbed by the elastic deformation; therefore, the viscosity and elasticity co-existed.

Figure 10 indicates that the viscoelasticity of the intercalated polymer was better than that of high-molecular-weight polyacrylamide, which was attributed to the unique molecular structure of the intercalated polymer. In addition, the electrostatic interaction and hydrogen bonding between the polymer molecular segment and the stripping layer strengthened the hydrophobic association between the original molecules in the bentonite, forming a more complex and stable three-dimensional spatial network structure.

#### 2.3.4. Salt Resistance

**Figure 11:**
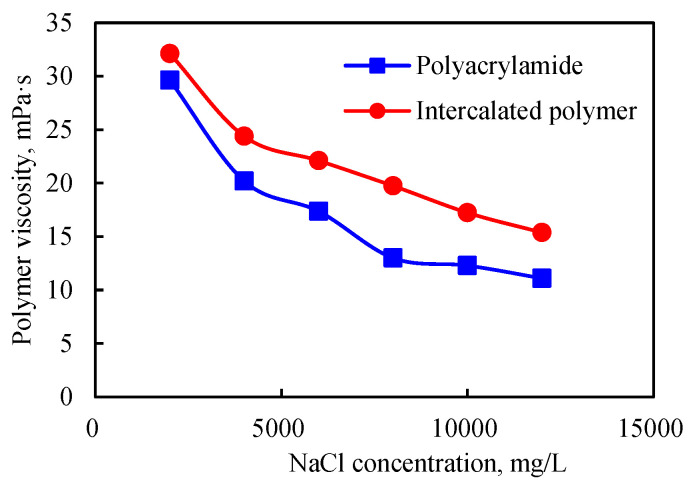
Salt resistance of the intercalated polymer.

When the concentration of the inorganic salt, i.e., NaCl, increased, the viscosity of the intercalated polymer solution decreased. For instance, when the inorganic salt concentration was 2000 mg/L, the viscosity of the polymer solution was about 32 mPa·s (Figure 11). Furthermore, the decrease in viscosity of the solution was lower than that of the polyacrylamide solution, indicating improved salt resistance for the polymer incorporated in the clay interlayer space.

When the silicate layer was dispersed in the matrix, intercalated polymers could exchange cations from the surface and arrange around the silicate surface via diffusion. When the salinity increased, the diffused double electric layer was compressed, and the electrical properties were reduced on the edges and surfaces, leading to the silicate layer’s edge connection and edge connection, resulting in a spatial structure and small viscosity loss. For polyacrylamide, when the salinity increased, the cations inhibited the dissociation of polymer molecules. The molecular chain expansion by the -COO- repulsion force was weakened, and the polymer wire group was greatly weakened, decreasing the polymer solution’s viscosity.

#### 2.3.5. Thermal Stability

The thermal stability evaluation results are shown in Table 3.

When the polymer concentration was constant, the viscosity of the polymer solutions first increased and then decreased. After 60 days of aging, the viscosity of the intercalated, high-molecular-weight solutions and salt-resistant polymers were 131.8, 90.3, and 98.3 mPa·s, respectively, whereas the viscosity retention rates were 89.1, 74.0, and 59.0%, respectively. Compared with the high-molecular-weight and salt-resistant polymers, the viscosity retention of the intercalated polymer increased by 40.1% and 15.1% after treatment at 85 °C for 60 days. Since a known amount of inorganic compound was used to prepare the intercalated nanocomposite, we can claim that the resulting organoclay’s thermal stability was significantly enhanced compared with conventional high-molecular-weight polyacrylamide, suggesting that the intercalated polymer solution displayed enhanced viscosity stability. The bentonite had the rigidity and thermal stability of inorganic materials. In addition, the polymer and bentonite lamellar structure formed a uniform three-dimensional network structure covering the entire system, enhancing the intercalated polymer’s stability.

## 3. Conclusions

(1) An intercalated polymer was synthesized using a low-temperature composite initiation system while controlling the polymerization temperature, clay mineral content, initiator, and chain extender concentration. The optimal synthesis conditions were a clay content of 10.7%, preparation temperature of 11 °C, initiator concentration of 2.5 × 10^−4^ mol/L, and chain extender concentration of 5%.

(2) The intercalated polymer structure was characterized. The IR results showed that the polymer was successfully added to the nanocomposite, and TEM analysis revealed that the intercalated polymer exhibited intercalated or exfoliated structure characteristics.

(3) Compared with the high-molecular-weight polymer, the intercalated polyacrylamide/clay nanocomposite possessed a better thickening effect, and the average viscosity increased by 48.9% with good viscoelasticity and better salt resistance and thermal stability. In addition, the viscosity retention at 85 °C for 60 d increased by 40.1%. The thickening capacity and thermal stability were also superior to salt-resistant polymers, increasing up to 16.0%, with a viscosity retention rate of 15.1%.

The new intercalated polyacrylamide/clay nanocomposite exhibited improved viscosity, salt resistance, and thermal stability, and its performance was close to or better than that of the salt-resistant polymer; however, the cost was lower, demonstrating good application prospects in the field of petroleum exploitation.

## 4. Materials and Methods

### 4.1. Instruments

An Agilent 5500 atomic force microscope, Hitachi EM-1200EX transmission electron microscope, Shimadzu FI-IR8DX infrared spectrometer, Brookfield DV-II rotational viscometer, Anton Paar MCR301 rheometer, Ubbelohde viscometer, and IKA T–25 high-speed dispersion machine were the main instruments used.

### 4.2. Methods

#### 4.2.1. Synthesis of the Intercalated Polymer/Clay Nanocomposite

(1) The inorganic clay mineral was dispersed in deionized water with a mass concentration of 3.85%–16.7% and stirred to form a homogenous suspension. (2) A weighed acrylamide monomer was added under high-speed stirring at 10000 r/min to produce an aqueous solution of the acrylamide-modified inorganic clay mineral. Then, the solution was transferred to a ground-glass flask. (3) Then, 0.10 g/L urea and SS-1 (solubilizer) at a mass ratio of 1:1 were added to the flask, along with 0.004 g/L complexing agent. (4) The flask was then placed in the water bath at a constant temperature and purged with nitrogen for 20 min. After removing the air, a known concentration of initiator was added. The ferrous sulfate/isopropyl benzene hydroperoxide redox system was used as an initiator, the molar ratio of FeSO_4_·7H_2_O to isopropyl benzene hydroperoxide was 1:9, and the final concentration was 1.0 × 10^−4^~3.0 × 10^−4^ mol/L. The nitrogen purging continued for another 3 min, and the flask was sealed. The polymerization conditions were monitored and the temperature variation was recorded. (5) After 4 h, the resulting gel pieces were removed and granulated. The granules were mixed with sodium hydroxide solution and dried at 90 °C for 1.5 h. Finally, the intercalated polymer sample was dried, pulverized, and sieved.

#### 4.2.2. Synthesis of the Intercalated Polymer/Clay Nanocomposite

The polymer’s viscosity and average molecular weight were measured using a Ubbelohde viscometer. The viscosity and average molecular weight of the polymer were determined as follows:

(1) $$[\eta ]=k{\bar{M_{v}}}^{{}^{\alpha }}$$

[*η*] is the intrinsic viscosity; *k* and *α* are constants related to temperature and solvent, respectively; and $\bar{M_{v}}$ is the viscosity average molecular weight of the polymer.

The inner diameter of the Ubbelohde viscometer was 1.36 mm. A 1 mol/L sodium nitrate solution was used as the solvent. At 30 °C, *k* = 3.73 × 10^−4^ and α = 2/3. The mass concentration of the polymer solution was 0.1%.

For low-concentration polymer solutions, the viscosities of the pure solvent and polymer solution were approximately equal, and the following formula can be used to calculate the intrinsic viscosity [*η*]:

(2) $$\left[\eta \right]=\frac{1}{2c}(\frac{\eta -\eta_{0}}{\eta_{0}}+\ln\frac{\eta }{\eta_{0}})=\frac{1}{2c}(\frac{t-t_{0}}{t_{0}}+\ln\frac{t}{t_{0}})$$

where *c* is the number of grams of polymer in 100 mL, *η*_0_ is the solvent viscosity, *η* is the polymer solution viscosity, *t*_0_ is the solvent outflow time, and *t* is the polymer solution outflow time.

#### 4.2.3. Thickening Test

A Brookfield DV-II rotary viscometer was used to test the apparent viscosity of the solution at 30 °C and 7.3 s^−1^.

The thickening performance of a 1200 mg/L polymer solution prepared via injected water, mixed water, and formation water was compared. The formation water was simulated water from Daqing Oilfield, with a total salinity of 4012.7 mg/L, and each 1000 g solution contained 0.2193 g Na_2_SO_4_, 0.1761 g KCl, 0.1569 g CaCl_2_, 0.2188 g MgCl_2_, and 3.2416 g NaHCO_3_.

The injected water was mixed with clean water and simulated formation water at a volume ratio of 1:5, and the salinity was 729.3 mg/L. The mixed water was a 1:3 mixture of injected water and simulated formation water, with a salinity of 3191.8 mg/L.

#### 4.2.4. Salt Resistance Test

Different concentrations of NaCl solutions were prepared in deionized water. NaCl solution was used to prepare 800 mg/L of polymer solution, and then a Brookfield DV-II rotary viscometer was used to test the apparent viscosity of the solution at 30 °C and 7.3 s^−1^.

#### 4.2.5. Thermal Stability Test

The thermal stability of the intercalated polymer and polyacrylamide in simulated wastewater at Daqing Oilfield was determined. Polymer solutions at 2000 ppm concentration were prepared in 50 mL colorimetric tubes and placed in a drying oven at 85 °C. Samples were withdrawn at regular intervals, and the viscosity was measured at 30 °C and 7.3 s^−1^.

## Figures and Tables

**Table 1:** Thickening effect of the intercalated polymer in water.

Polymer Type	Apparent Viscosities of Polymers at Different Concentrations, mPa·s				
	800 mg/L	1000 mg/L	1200 mg/L	1400 mg/L	1600 mg/L
Intercalated	59.4	106.6	148.0	221.6	292.9
High molecular weight	40.3	65.4	122.0	139.6	190.3
Salt-resistant	54.5	79.7	166.6	185.3	227.0

**Table 2:** Thickening effect of intercalated polymer in water with different salinities.

Polymer Type	The Viscosities of Polymer Solution Prepared with Different Types of Water, mPa·s			Viscosity Loss, %
	Injected Water	Mixed Water	Formation Water	
Intercalated	148.0	60.6	43.2	70.8
High molecular weight	122.0	44.7	31.2	74.4
Salt-resistant	166.6	59.1	38.4	77.0

**Table 3:** Thermal stability of the intercalated polymer.

Polymer Type	Placing Time (days)								Viscosity Retention Rate (%)
	Initiation	2	5	10	20	30	40	60	
Intercalated	148.0	221.0	195.3	163.0	150.6	142.6	142.3	131.8	89.1
Salt-resistant	122.0	118.3	117.3	111.0	103.6	93.7	92.7	90.3	74.0
High molecular weight	166.6	175.3	143.3	131.6	116.3	111.0	104.6	98.3	59.0

## Data Availability

The data presented in this study are available upon request from the corresponding author.
